# The Physiology or Mechanism of Blushing; Illustrative of the Influence of Mental Emotion on the Capillary Circulation; with a General View of the Sympathies, and the Organic Relations of Those Structures with Which They Seem to Be Connected

**Published:** 1839-07

**Authors:** 


Art. X.—*The Physiology or Mechanism of Blushing; illustrative of the Influence of mental Emotion on the capillary Circulation; with a general view of the Sympathies, and the organic relations of those Structures with which they seem to be connected.* By Thomas H. Burgess, m.d. &c.—*London*, 1839. 8vo, pp. 202.

We opine that our Review does not find many readers among the fair sex; its character is too profound for the Lady Bountiful who smatters in medical subjects; and it will scarcely furnish much light reading to those who live on periodical literature. To whom else to recommend a work on the Physiology of Blushing, would have sorely perplexed us, if we had not found upon examination that the title but faintly shadows forth the character and scope of its contents. To be sure, the first sentence of Chapter I. is in the true young-lady style of unmeaning sentimentality —"Blushing may be styled the poetry of the soul!" But our author mends upon acquaintance; and out of his two hundred pages there are really some (though, by the way, *not* upon his chosen subject,) which contain better matter than this promises. As our readers will probably feel some curiosity to know how he treats of so novel a subject, we shall present them with a sketch of the table of contents, which want of room only prevents our transferring to our pages, as proving how admirable a specimen the book is of that interesting genus of literary productions which treat de omnibus rebus, &c.

The first section is devoted to the *Natural History* of Blushing, of which the "*Poetry*" of this phenomenon, with "Virgil's imagery," and "Homer's description of 'Pale Fear,'" constitutes the first chapter. The Sensibility of Plants, that of the lower Animals and of Man, are then discussed. The motions of the former are considered as being either the "effect of the vital principle in a state of concentrated action," or as depending on the "germ of true sensation;" to which last opinion the author inclines. Sensibility in man is defined to be "that state of feeling which draws a distinction between right and wrong!" Then follows a chapter on Blushing as an Evidence of Design; and next a disquisition on the varieties of the human race. In Chapter V., the varieties of the Blush are classified, as into the True Blush, the Blush of Feeling, &c.; and in the last chapter of the section, the important question is considered whether the idiot, the lunatic, or the intoxicated man can blush; and this is decided in the negative. Section ii. is concerned about the *Anatomy* of Blushing; and here we have one chapter on the Nervous System, another on the Capillary Vessels, and a third upon the Skin, in which the structure and functions of these parts are systematically treated of. In the third Section is discussed the *Mechanism* of Blushing. In this part of the work, after the "reflected movements of sympathy," the "Epileptic Aura," the identity between electricity and nervous influence, and a few other equally disconnected subjects have been dismissed, the author proceeds to establish his peculiar (?) doctrine,—namely, that the "Blush-exciting impulse" is conveyed from the brain to the stomach and solar plexus, and along the ganglionic nerves to the capillaries,— which we believe to be the doctrine usually entertained by physiologists at the present time, (except as to the necessity of the transmission of the impulse to the solar plexus, which we take leave to doubt,) and which it scarcely required two hundred octavo pages to establish.

This abridgment but slightly justifies what we have said of the discursive character of the book; however, if our readers are inclined to judge for themselves, we venture to predict that they will derive some amusement if not much instruction from the treatise. The conclusion, however, is too rich to be overlooked. What will the following subjects think of their fellowship?—"Moral and Physical Treatment of the Mental Emotions arising from Diseased Sensibility—Early Moral Training and its advantages—Physical Treatment—The salutary influence which gymnastic exercises exert on the Intellectual and Moral powers—Pernicious effects of Boarding-school Physical Education—Gymnastics recommended by Herodicus, Hippocrates, Galen, Celsus, Sanctorius, Van Swieten, *Macartney*—Cultivation of Gymnastic Education in Paris—Gymnasium of MM. Pravaz and Jules Guerin."

One word to the author of the work we have been so freely criticising. Let him choose for his next attempt a better subject; let him cultivate precision of language, and closeness of thought; and discard or sparingly use the flowery ornaments which garnish his present composition. Then if the desire of authorship should still be strong within him, he may stand a chance of attaining at least a respectable station as a writer, and may please others besides the superficial and trivial-minded, to whom alone, we fear, his present work will recommend him.

